# Alcohol, the Brain, and Behavior: Mechanisms of Addiction

**Published:** 2000

**Keywords:** neurobehavioral theory of AODU (AOD [alcohol or other drug] use, abuse, and dependence), brain, synapse, neuron, cell signaling, intracellular messengers, protein kinases, phosphorylation, AOD tolerance, AOD withdrawal syndrome

## Abstract

The actions of alcohol that cause intoxication, initiate and maintain excessive drinking behavior, and promote relapse during abstinence occur primarily in the brain. A thorough understanding of alcohol’s effects on the mechanisms underlying brain function is essential to develop and improve alcoholism prevention and treatment strategies. This article is not an exhaustive overview of alcoholism neurobiology, but a sampling of the more significant recent advances in the field.

The specific mental processes thought to underlie the development of alcoholism and its manifestations involve functions such as learning, attention, emotion, and cognition. The normal brain must orchestrate these functions simultaneously to perceive the environment, assess the significance of environmental stimuli in terms of survival, and initiate behavioral reactions. These activities require efficient communication among different regions of the brain and at multiple levels within those regions. This article considers alcohol’s effects on three levels of communication within the brain: (1) the synaptic level, involving information transfer between individual nerve cells (neurons); (2) the systems level, representing the integrated activity of different brain regions; and (3) the intracellular level, comprising signaling processes that occur within neurons.

## Alcohol at the Synapse: Modifying Communication Between Neurons

Within the brain, each neuron may communicate with many other neurons. Information is transferred by chemical messengers called neurotransmitters, which are released by one neuron and then bound by specialized proteins called receptors embedded in the outer membrane of another neuron. The tiny gap between communicating neurons is called a synapse. There are many neurotransmitters, each of which binds to a particular receptor. However a given receptor may exist in multiple subtypes. Each subtype may produce a different response to the same neurotransmitter, accounting for multiple effects of the same neurotransmitter in different brain regions or even in different locations on the same neuron (Weiner et al. 1997). Although a given neuron can release only one or two neurotransmitters it may possess different receptors. Thus, a neuron’s response to information from other neurons depends on complex interactions of potentially conflicting messages arriving simultaneously (Charness 1990).

One of the most powerful effects of alcohol is to reduce the pace of brain activity in part by (1) decreasing the excitatory actions of the neurotransmitter glutamate at the NMDA subtype of glutamate receptor^1^ and (2) boosting the inhibitory actions of the neurotransmitter gamma-aminobutyric acid (GABA) at the GABA_A_ receptor (Diamond and Gordon 1997). These actions are among the reasons that alcohol is often thought of as a depressant.

The NMDA and GABA_A_ receptors are linked to ion channels; that is, they function by opening a pore through the cell membrane to allow specific ions (electrically charged atoms) to enter the cell and affect the cell’s electrical balance (Harris 1999). Other neurotransmitters of interest to alcohol researchers include dopamine, serotonin (5–HT), and a family of substances called opioid peptides. These neurotransmitters interact with their receptors to modulate the activity of the neuron on which they reside.

Dopamine’s role in coordinating the execution of complex motor activities has long been recognized. Dopamine also appears to play a major role in motivational behavior (i.e., the pursuit of rewarding stimuli). Alcohol administration causes release of dopamine in a brain region (the nucleus accumbens) that is a key member of a group of linked structures associated with the development of addiction (Rassnick et al. 1992; Brodie and Pesold 1999).

Opioid peptides are a class of neurotransmitters that produce physiological effects similar to those of morphine and heroin. In humans, opioid peptides interact with other neurotransmitters to influence a broad range of physiological functions, including the control of pain. High blood levels of certain opioid peptides have been correlated with feelings of euphoria. Alcohol consumption affects the activity of opioid peptides, which in turn appears to increase the rewarding effects of alcohol (Roberts et al. 2000). The medication naloxone, which inhibits the function of opioid receptors, blocks the release of dopamine in the nucleus accumbens and has been shown to suppress alcohol consumption by laboratory animals (Benjamin et al. 1993).

Serotonin is involved in the regulation of mood, sleep, body temperature, appetite, and a host of other physiological functions. Experiments in which mice have been genetically altered to lack specific serotonin receptor subtypes have suggested a role of serotonin on drinking levels. Alcohol-induced activation of specific serotonin-receptor subtypes can stimulate dopaminergic activity in the nucleus accumbens, potentially contributing to alcohol’s rewarding effects. Other serotonin receptors have potential roles in tolerance,^2^ withdrawal,^3^ and intoxication (Valenzuela 1997).

## Alcohol and Neuronal Circuits: Detours on the Information Highway

Since the discovery in the late 1980s that alcohol at concentrations capable of producing intoxication in humans can inhibit the excitatory effects of the NMDA receptor and enhance the inhibitory function of the GABA receptor, much alcohol research has focused on identifying other specific receptors and ion channels that may be affected by alcohol. However, unlike most illicit drugs of abuse, alcohol does not have a specific neurotransmitter binding site in the brain. Moreover, the complex behaviors associated with alcohol use cannot be attributed to a limited number of specific chemical interactions. This realization sparked a closer look at alcohol’s effects on pathways of neuronal communication that integrate the activities of multiple brain regions.

### Reinforcement and Neuroadaptation

Two major processes that contribute to development of addiction are reinforcement and neuroadaptation. Reinforcement is when a rewarding stimulus (e.g. alcohol and other drug [AOD] induced euphoria) or relief of an unpleasant state (e.g., anxiety) increases the probability of a behavioral response (e.g., AOD use). Neuroadaptation refers to compensatory adjustments whereby the brain attempts to continue normal function despite the presence of alcohol. Occurring essentially simultaneously, reinforcement and neuroadaptation appear to underlie both the initial, short-term (acute) response to a drug and the establishment of the long-term (chronic) craving that characterizes addiction. Some neuroadaptive changes may be permanent, producing the persistent sense of discomfort that may lead to relapse long after a person stops drinking (Koob et al. 1993).

A common manifestation of neuroadaptation is the occurrence of an acute withdrawal syndrome following the abrupt cessation of a bout of heavy drinking. In response to the continued presence of alcohol, compensatory mechanisms attempt to overcome alcohol’s inhibition of NMDA receptors by increasing overall NMDA function (upregulation). When alcohol leaves the synapse, the combination of upregulated excitatory transmission and downregulated inhibitory transmission results in the brain hyperexcitability characteristic of the acute withdrawal syndrome (Littleton 1998). Some brain damage occurs during acute alcohol withdrawal, and the severity of symptoms increases after repeated withdrawal episodes (Becker 1998). Withdrawal triggers the body’s stress response, leading to elevated levels in the blood of the stress hormone cortisol. Excessive cortisol levels can kill neurons in the hippocampus, increase the risk of infectious diseases, alter energy metabolism, and promote disorders of mood and intellect (Adinoff et al. 1998).

Neuroadaptation is usually thought of in terms of counteradaptation—processes such as tolerance that are initiated to oppose the acute effects of drugs. However, neuroadaptation also includes sensitization, an *increased* response to a drug effect following repeated drug administration. If sensitization induces increased AOD consumption (as in the motivational state called “wanting” or craving, discussed later), it may contribute to addiction. Sensitization may be more likely to occur with intermittent, repeated exposure to AODs, whereas tolerance is more likely to occur with continuous exposure (Robinson and Berridge 1993).

Acute alcohol withdrawal includes motivational effects (Koob and LeMoal 1997). The neurological structures associated with the reinforcing actions of alcohol and other drugs may involve a common neural circuitry that forms a separate entity within the basal forebrain, the extended amygdala (Alheid and Heimer 1988). The term extended amygdala refers to a complex of several small structures near the base of the front of the brain that are similar in cell structure, function, and neural connectivity. This system has extensive connections to brain regions that play central roles in reinforcement and reward (Diamond and Gordon 1997).

Rats trained to self-administer alcohol during withdrawal show neurochemical and neuropharmacologic changes indicative of alterations in GABAergic, dopaminergic, and serotonergic function in specific components of the extended amygdala. One key structure encompassed by the extended amygdala is the nucleus accumbens, which has long been implicated in the rewarding properties of AODs. Other investigations have demonstrated selective activation of dopaminergic transmission in the shell of the nucleus accumbens in response to acute administration of virtually all major drugs of abuse (Tanda et al. 1997).

## Alcohol and Molecules: Sabotaging the Communications Infrastructure

Coordinated interneuronal communication can help account for relatively transient features of alcoholism, such as acute tolerance, physical withdrawal symptoms, and the initiation of reinforcement, which can lead to dependence. However, studying these processes alone does not provide a complete understanding of longer-term manifestations of alcoholism, such as the uncontrolled craving that may contribute to relapse years after cessation of drinking. The mechanisms underlying alcohol’s chronic effects involve processes called intracellular signaling, the cell’s internal biochemical response to receptor activation by extracellular chemical messengers.

### Some Aspects of Normal Signaling

Intracellular signaling helps provide a link between the cell’s initial response to alcohol and persistent alterations in neuronal function similar to the processes involved in memory. These processes may involve the activation of genes that direct the synthesis of (i.e., express) specific proteins such as components of receptors or structural elements of the outer neuronal membrane. Such changes can strengthen information flow between neurons, helping close the loop between neurotransmitter activation and alcohol-related behavior.

Intracellular signaling comprises a complex network of mutually interacting processes. Among the common themes that emerge is the regulatory role of protein-phosphorylating enzymes (e.g., protein kinases). Phosphorylation is the attachment of a phosphate group (a cluster of phosphorus and oxygen atoms) to a molecule. Phosphorylation can activate regulatory enzymes (e.g., G proteins), modify the function of ion channels, or activate transcription factors that initiate the expression of specific genes (Diamond and Gordon 1997).

Many signaling pathways involve molecules called second messengers. These molecules may regulate short-term events (e.g., ion channel activity and neurotransmitter release) as well as longer-term processes (e.g., synaptic plasticity, memory, and learning). Some G proteins may influence shifts in alcohol sensitivity following chronic alcohol exposure by affecting the function of calcium-specific ion channels. Calcium itself can be a second messenger, and is required to stimulate many neuronal activities, including the release of neurotransmitters.

### Alcohol’s Effects on Protein Phosphorylation

Some protein kinases are located within the neuron, whereas others are attached to the inner surface of the neuronal membrane, where they chemically modify the structure of receptors to help regulate their function. Certain kinases appear to influence alcohol’s effects on various NMDA and GABA receptor functions (Diamond and Gordon 1997).

The role of kinases in alcoholism has been studied using the techniques of genetic engineering. Null mutant, or knockout (KO), mice are made by replacing a normal gene with an inactive gene. Transgenic mice are created by permanently adding a foreign gene that may function differently than the animal’s natural gene.

Schuckit (1998) has suggested that low initial sensitivity to alcohol’s behavioral effects among sons of alcoholics is associated with increased risk of future alcoholism, possibly mediated in part by more rapid development of tolerance (Schuckit 1998). Knockout of a specific kinase that interacts with GABA_A_ receptors in mice produces results consistent with Schuckit’s observations in humans (Weiner et al. 1997).

Knockout of a different kinase in mice inhibits the function of hippocampal NMDA receptors, impairs the development of acute tolerance to alcohol, increases sensitivity to alcohol-induced sedation, and impairs spatial learning. Some evidence indicates that this kinase may modulate the activities of both NMDA and GABA_A_ receptors to determine alcohol sensitivity (Yagi 1999).

## Footnotes

1 So called because the synthetic chemical *N*-methyl-d-aspartate (NMDA) also can activate this receptor subtype.

2 Tolerance, or the reduction in a drug effect after repeated use, may stimulate a user to take increasing doses of the drug in an attempt to reexperience its initial effect.

3 Acute withdrawal syndrome begins from 6 to 48 hours after the last drink and may include tremors, elevated blood pressure, increased heart rate, and seizures. Alcohol and other drug (AOD) withdrawal also includes changes in mental state (anxiety, depression, and craving) that may outlast the physiological symptoms and motivate renewed AOD consumption.
